# Prevalence and associated factors of common mental disorders among Ethiopian migrant returnees from the Middle East and South Africa

**DOI:** 10.1186/s12888-017-1310-6

**Published:** 2017-04-19

**Authors:** Kassahun Habtamu, Abebaw Minaye, Waganesh A. Zeleke

**Affiliations:** 1 School of Psychology, College of Education and Behavioral Studies, Addis Ababa University, P.O. BOX: 150588, Addis Ababa, Ethiopia; 2 Department of Counseling, Psychology and Special Education, Duquesne University, 209-C Canevin Hall, 600, Forbes Avenue, Pittsburgh, PA 15282 USA

**Keywords:** Migration, Common mental disorders, Ethiopia, Middle East countries, South Africa, Migrant returnees, Prevalence

## Abstract

**Background:**

Ethiopian migrants to the Middle East and South Africa experience a range of problems at various stages of their migration including overwork, sleep deprivation, denial of food, emotional abuse, difficulty adapting to the host culture, salary denial, sexual abuse, labor exploitation, confiscation of their travel documents, confinement, denial of medication, lack of access to legal service and degrading attitude by employers, traffickers and smugglers. These experiences can be associated with different types of mental disorders. This study sought to determine the prevalence of common mental disorders (CMD) and socio-demographic and other migration related associated factors among Ethiopian migrant returnees from the Middle East and South Africa.

**Method:**

A cross-sectional study was conducted using non-probability (i.e. purposive, availability and snowball) sampling techniques. Migrant returnees (*n* = 1036) were contacted individually at their homes in eight high prevalent immigrant returnee locations in Ethiopia. Common mental disorders were assessed using the self-reporting questionnaire (SRQ-20) and a structured questionnaire was employed to collect data on socio-demographic and migration related characteristics. Data were analyzed using descriptive statistics, univariate logistic regression, and multivariable logistic regression.

**Results:**

The prevalence of CMD among migrant returnees was found to be 27.6%. Highly prevalent specific CMD symptoms included headaches, poor appetite, being tired, sleeping problems, and feeling unhappy or nervous. Being originally from Amhara and Oromia regions, being Christian, being divorced, not receiving salary on time, not being able to contact family, unable to prepare for domestic labor abroad, lack of cross- cultural awareness, and lack of knowledge and skills for work were all important risk factors for CMD. Migrants experienced adversities at different stages of their migration which are associated with psychological distress and even to long term mental illnesses.

**Conclusions:**

CMD symptoms were found to be prevalent among Ethiopian migrant returnees. As pre-migration factors are associated with CMD symptoms, pre-departure training could be useful to mitigate the risk factors. Creating and routinely arranging mental health interventions and rehabilitation services are advisable for returnees who are screened for, or diagnosed with, mental health problems.

**Electronic supplementary material:**

The online version of this article (doi:10.1186/s12888-017-1310-6) contains supplementary material, which is available to authorized users.

## Background

Migration is a universal phenomenon; it has been part of the human history through the ages and across nations [1, 2]. Migration is qualitatively and quantitatively a highly heterogeneous process [3]; it may involve one individual; a group; a family or a group of families [4]. Ethiopia is one of the big sources of migrant flow particularly to the Middle East and South Africa [3, 5]. Migration from this African nation has increased in scope and magnitude recently, largely facilitated by the ongoing globalization, and the diffusion of modern communications [6]. There are an estimated 1.5 million Ethiopian migrants worldwide, and every year close to 120, 000 Ethiopians migrate [7]. Another evidence of this huge migration is the deportation of 170,000 Ethiopians from Saudi Arabia, in only 5 months from October 2013 to February 2014 [8].

Some studies indicate that migrant populations are mentally healthier than native populations. For instance, Stillman, McKenzie and Gibson [9] found that migration lead to improvements in mental health in New Zealand. Similar evidence for better mental health of immigrants was also found in a study conducted in Canada [10]. However, the majority of studies indicate elevated levels of various mental disorders among migrants [4, 11–14]. For example, Warfa and Colleagues [13] found migration to be associated with poor mental health among Somali refugees in the UK, while a meta-analysis of the incidence of schizophrenia in migrants shows increased rates of the disorder, particularly when migrants come from a developing country or from a predominantly black population [15]. Psychiatric morbidity has been found to be two to five times higher among women migrating as domestic workers to the different Middle Eastern countries than in the native populations there [16, 17].

Several hypotheses were put forward to explain the high rates of mental health problems among migrant groups. The most plausible explanation say that although the purpose of migration for most people is seeking a better quality of life, migration is a stressful life event where the stress compounds to the point of psychological disorder. [18]. According to Berry [19], migrants pass through acculturative stress, which results from the dilemma over how and whether to maintain their own identity and relationship with others, imposing changes in language, cognitive style, personality, identity, and attitude.

Generally, migrants face a variety of pre-migratory, migratory, and post- migratory stressors that can have lasting impacts on their mental health [20]. Chang-Muy and Congress [21] articulated that the migration precipitating factors in origin countries that induced the intention to migrate, the escape journey, and the relocation process to the destination are found to be shocking and often are associated with trauma (which is described as triple trauma).

Specific evidence on the mental health of Ethiopian migrants is scarce. Nevertheless, the few studies conducted on Ethiopian migrants residing in different countries and returnees from different Middle East countries [14, 22–24] show that Ethiopian migrants often have a variety of psychological problems [22, 23] as they experience diverse problems at the various stages of their migration [3, 25]. Some of the challenges include [22]: physical and emotional abuse while travelling; poor adaptation to the host culture; physical, emotional and sexual abuse at destination; overwork and salary denial; loss of identity and degrading attitudes of employers; very poor legal support; and lack of psychosocial support. Several studies showed that these challenges are strongly associated with different kinds of mental disorders, including depression, post-traumatic stress disorder and somatization [11, 26–30].

A few qualitative case studies have been conducted to explore the experiences and psychological problems of migrant domestic workers returned from different Middle Eastern countries. These studies revealed that at the tip of the cumulative oppressive experiences of migrants is mental distress [3, 22]. No large-scale epidemiological study that estimates the magnitude and risk factors for mental health problems among Ethiopian migrant returnees from the Middle East and South Africa has been conducted. Hence, this study investigates the magnitude of common mental disorders (CMD) among Ethiopian migrant returnees from the Middle East and South Africa and the associated socio-demographic and migration-related factors. Those migrated to the Middle East are mostly domestic workers, while those who migrated to South Africa engage in tasks such as street vender and work as daily laborers.

## Methods

A cross-sectional survey using non-probability sampling (availability, purposive and snowball sampling), was conducted to collect data from migrant returnees at 8 study sites in Ethiopia.

### Study sites

The selected study sites were chosen purposively based on high prevalence of migration. First, the four larger regions of the Federal Democratic Republic of Ethiopia, namely Oromia, Amhara, Southern Nations, Nationalities and People’s Region (SNNPR), and Tigray were selected as they constituted the larger base of migration from Ethiopia to the Middle East and South Africa. From the four regions, 6 hotspots were purposively selected based on the high prevalence of migration, namely South Wollo from Amhara, Eastern Zone from Tigray, Jimma and Arsi from Oromia and Hadiya and Butajira from SNNPR. In addition, two city administrations (Addis Ababa and Dire Dawa) were added making the total study sites to be 8.

### Participants

One thousand thirty six migrant returnees were selected through convenient, availability and snowball sampling. We used a door-to door method to approach and recruit participants. The inclusion criteria used included being a migrant returnee from one of the Middle East countries (including, but not limited to the Kingdom of Saudi Arabia, United Arab Emirates, Lebanon, Qatar, Jordan, Bahrain and Kuwait) or South Africa, having returned in the last six months, being18 years of age or older, being able to answer the interview questions in Amharic and being able to give verbal informed consent. Those in treatment for any mental illness or in acute physical illness were excluded from the study. The reason is that it is difficult to involve them and their mental health problem is self-evident and thus, we felt did not need to be measured.

### Assessment of the outcome and predictor variables

A structured questionnaire constructed by a team of researchers in different social science fields for a larger thematic research project was used to collect data on the socio-demographic and migration related characteristics of the participants. The questionnaire included several queries regarding the personal background characteristics of returnees (11 questions), background characteristics of returnees’ family (six questions), returnees’ experiences from the pre-migration to the post-return stages (42 questions) and questions related to the returnees’ attitudes towards migration (8 questions). The questionnaire was reviewed by both measurement and subject experts and pilot tested with respondents having similar attributes as the main study participants. Based on the pilot study, questions found to be less understandable were modified. As part of the questionnaire described above, the 20 items self-reporting questionnaire (SRQ-20) was included to assess common mental disorders. The SRQ-20 was developed by the World Health Organization (WHO) to improve detection of common mental disorders in primary health care settings in low and middle income countries (LMICs) [31]. Items in the SRQ-20 are single-clause questions, with a response format of yes or no, and easily administered in an interview format. The items included questions regarding somatic, anxiety and depressive symptoms, as well as questions about suicidal ideation and functional impact, present in the preceding 30 days. The 20 items version of the SRQ has been used and validated in several LAMICs. In Ethiopia, the SRQ-20 has been used in several facility-based and population-based epidemiological studies to screen depression and CMD. It has been validated for detection of depression and CMD in the primary healthcare setting, where evidence of criterion, convergent and construct validity were present [32, 33] (Additional file 1).

### Data collection procedures

To collect data for the study, seven supervisors were dispatched (one supervisor for each site except for Addis Ababa and Dire Dawa, where one supervisor was assigned to handle both sites). The supervisors were members of a larger thematic research project where this study was nested, and their roles were to train data collectors, oversee participant recruitment and data collection and checking and controlling data quality. A total of 14 data collectors (two for each site) with at least a first degree level training mainly in the social sciences were recruited. A two-days long training was provided for the data collectors on the purpose of the study, the contents of the data collection instruments, ethical matters, and on how to recruit and approach participants. Data collectors went door to door in areas where migrant returnees were available via the guidance of key informants in each locality. The data- collection process was closely followed-up by the supervisors.

### Data analysis

Data were checked for completeness and consistency by the field supervisors during data collection and by the first author after data entry and before data analysis began. Data entry and analysis were carried out using Statistical Packages for the Social Sciences (SPSS version 20). Data were entered into SPSS by trained and experienced data entry clerks. Descriptive statistics (frequency and percentage) were used to summarize the background characteristics of the participants. Descriptive statistics were also used to determine the prevalence of common mental disorders and examine the distribution of specific symptoms contained in SRQ-20 among the study population. Univairate and multivariable logistic regression models were fitted to assess the association of the outcome variable (CMD) with potential risk factors. These potential risk factors were selected a priori based on evidence from the existing literature and our theoretical assumption that these factors would be relevant for CMD. These potential demographic and migration related factors were incorporated in the survey questionnaire. Only factors that were associated with the outcome (CMD) in the univariate models were included in the corresponding multivariable model in order to limit the potential risk of over adjusting without compromising identification of potential predictors for the outcome. Odds ratios were used to determine the strength of associations in selected variables. The cut off for statistical significance was set at α = 0.05.

### Ethical considerations

The study design was reviewed and approved by a technical committee established by the Office of the Vice President for Research and Technology Transfer, Addis Ababa University. Approval was also obtained from the concerned offices in all the study sites. Concerned offices are those district level government offices mandated to work on migration and related issues. These offices include Labor and Social Affairs Office, Justice Office and the Police.

Participation was voluntary and verbal informed consent was obtained from all the participants. Verbal informed consent was preferred than written informed consent just to put respondents at ease since people are not confortable to put their signature on paper in the Ethiopian socio-cultural context. Informants were informed about the purpose of the study and they were told that they could withdraw at any time from the study and cease responding to any question they felt uncomfortable. Information obtained from all the participants was anonymized and confidentiality was assured throughout the data collection process.

## Results

### Socio-demographic characteristics of participants

A total of 1036 migrant returnees from 8 research locations participated in this study. For details of the socio-demographic characteristics of the participants, see Table 1. Over half of the participants (55.1%) were women. In terms of religion, 50% identified themselves as being Muslim and 48.1% Christian. The mean age of the participants was 28 years (Range: 18–63). Participants were from diverse ethnic groups, including Oromo, Amhara, Gurage, Tigre, and Hadiya. The majority of the participants had either a primary (35%) or secondary (44%) level of education. Four hundred fifty-six (49.5%) of the participants were from rural areas.

**Table 1:** Background characteristics of the study participants

Characteristics		Number^a^	Percent
Sex (*N* = 1027)			
	Male	461	44.9
	Female	566	55.1
Age (years)	Mean (standard deviation)	28 (6.6)	
Religion (*N* = 1019)			
	Muslim	519	50.9
	Orthodox	298	29.2
	Protestant	175	17.2
	Catholic	17	1.7
	Other	10	1.0
Ethnicity (*N* = 849)			
	Oromo	166	19.6
	Amhara	233	27.4
	Tigre	86	10.1
	Gurage	166	19.6
	Hadiya	131	15.4
	Kembata	16	1.9
	Other	51	6.0
Region (*N* = 1015)			
	Tigray	145	14.3
	Amhara	204	20.1
	Oromia	264	26.0
	SNNPR	351	34.6
	Addis Ababa	40	3.9
	Dire Dawa	11	1.1
Educational status (*N* = 1025)			
	Cannot read and write	65	6.3
	Read and write only	77	7.5
	Grade 1–4	89	8.7
	Grade 5–8	267	26.0
	Grade 9–10	321	31.3
	Grade 11–12	132	12.9
	Certificate/college diploma	59	5.8
	First degree or above	15	1.5
Marital status (*N* = 1005)			
	Married	448	44.6
	Single	510	50.7
	Divorced	44	4.4
	Separated/widowed	3	0.3
Residence before migration (*N* = 922)			
	Rural	456	49.5
	Urban	466	50.5
Source of family income (*N* = 955)			
	Farming	628	65.8
	Trading	167	17.5
	Government employment	106	11.1
	Other	54	5.7

^**a**^The fluctuation in the total number of respondents in each demographic variable is due to missing data

### Prevalence of common mental disorders

The prevalence of common mental disorders among Ethiopian migrant returnees from Middle Eastern countries and South Africa was found to be 27.6% (*N* = 260/942). When we look closely at the prevalence of specific symptoms of CMD (see Table 2), the following were found to be highly prevalent: headaches (40.6%), poor appetite (39.4), fatigue (35.8%), difficulty sleeping (36.9%), feeling unhappy (37.6%), and feeling nervous or tense (32%), whereas symptoms like hand tremors (14.6%), trouble thinking clearly (19.3%), suicidal ideation (15%), problems with decision making (20%) and functional impairment (19.9%) were relatively less common.

**Table 2:** Prevalence of common mental disorders and distribution of specific symptoms

Items	Response categories		
	Yes N (%)	No N (%)	Total N (%)
Do you often have headaches?	404 (40.6)	591 (59.4)	995 (100.0)
Is your appetite poor?	393 (39.4)	604 (60.6)	997 (100.0)
Do you sleep badly?	368 (36.9)	630 (63.1)	998 (100.0)
Are you easily frightened?	274 (27.6)	718 (72.4)	992 (100.0)
Do your hands shake?	146 (14.6)	851 (85.4)	997 (100.0)
Do you feel nervous, tense or worried?	319 (32.0)	678 (68.0)	997 (100.0)
Is your digestion poor?	256 (25.7)	740 (74.3)	996 (100.0)
Do you have trouble thinking clearly?	192 (19.3)	804 (80.7)	996 (100.0)
Do you feel unhappy?	373 (37.6)	620 (62.4)	993 (100.0)
Do you cry more than usual?	238 (23.9)	759 (76.1)	997 (100.0)
Do you find it difficult to enjoy your daily activities?	275 (27.6)	721 (72.4)	996 (100.0)
Do you find it difficult to make decisions?	199 (20.0)	794 (80.0)	993 (100.0)
Is your daily work suffering?	198 (19.9)	797 (80.1)	995 (100.0)
Are you unable to play a useful part in life?	209 (21.0)	785 (79.0)	994 (100.0)
Have you lost interest in things?	259 (26.1)	734 (73.9)	993 (100.0)
Do you feel that you are a worthless person?	191 (19.2)	803 (80.8)	994 (100.0)
Has the thought of ending your life been on your mind?	149 (15.0)	843 (85.0)	992 (100.0)
Do you feel tired all the time?	351 (33.9)	642 (64.7)	993 (100.0)
Do you have uncomfortable feelings in your stomach?	274 (27.6)	719 (72.4)	993 (100.0)
Are you easily tired?	356 (35.8)	639 (64.2)	995 (100.0)
CMD^a^ cases (with cut-off point ≥8)	260 (27.6)	682 (72.4)	942 (100.0)

^**a**^Common mental disorders

### Factors associated with common mental disorders

Both univariate (Table 3) and multivariable (Table 4) logistic regression models are presented. In the univariate model, being Muslim, being from SNNPR, and having a current health status the same as or better than it was before migration were negatively associated with CMD; and all these associations were statistically significant. In the same model, being from Oromia or Addis Ababa regions, being divorced, urban residence before migration, receiving salary sometimes or not at all during post-migration, having less knowledge on the type and nature of work in the destination country, and having no cross-cultural awareness and knowledge of the lifestyle of the destination country before leaving were positively and significantly associated with CMD. However, female gender, being of Protestant and Catholic religions, being from Amhara region, higher educational status, having break time during their migration work and being allowed to contact family and to visit friends/relatives were not associated with CMD.

**Table 3:** Univarite logistic regression analysis for the association of socio-demographic and migration-related factors with CMD

Variables		Yes N (%)	No N (%)	Odds ratio	95% CI	*P*-value
Sex						
	Male	111 (26.4)	310 (73.6)	1.0		
	Female	148 (28.7)	367 (71.3)	1.13	[0.84, 1.50]	0.42
Religion						
	Orthodox	92 (35.0)	171 (65.0)	1.0		
	Muslim	112 (23.0)	376 (77.0)	0.55	[0.40, 0.77]	<0.01
	Protestant	46 (30.7)	104 (69.3)	0.82	[0.54, 1.26]	0.37
	Catholic	4 (26.7)	11 (73.3)	0.68	[0.21, 2.18]	0.51
Region						
	Tigray	40 (29.6)	95 (70.4)	1.0		
	Amhara	54 (27.8)	140 (72.2)	0.92	[0.56, 1.49]	0.72
	Oromia	91 (39.6)	139 (60.4)	1.56	[0.99, 2.45]	<0.05
	SNNPR	52 (16.2)	269 (83.8)	0.46	[0.29, 0.74]	<0.01
	Addis Ababa	16 (47.1)	18 (52.9)	2.11	[0.98, 4.55]	<0.05
Education						
	No education	14 (24.1)	44 (75.9)	1.0		
	Reading and writing	24 (39.3)	37 (60.7)	2.04	[0.92, 4.49]	0.08
	Grade 1 to 4	18 (20.9)	68 (79.1)	0.83	[0.38, 1.84]	0.65
	Grade 5 to 8	60 (24.2)	188 (75.8)	1.00	[0.51, 1.96]	0.99
	Grade 9 to 10	80 (27.8)	280 (72.2)	1.21	[0.63, 2.33]	0.57
	Grade 11 to 12	40 (33.3)	80 (66.7)	1.57	[0.77, 3.20]	0.21
	Certificate/Diploma	18 (32.1)	38 (67.9)	1.49	[0.65, 3.39]	0.34
	First degree and above	6 (42.9)	8 (57.1)	2.36	[0.70, 7.96]	0.17
Marital status						
	Married	106 (25.6)	310 (74.5)	1.0		
	Never married	132 (28.7)	328 (71.3)	1.18	[0.87, 1.59]	0.286
	Divorced	16 (44.4)	20 (55.6)	2.34	[1.17, 4.68]	<0.05
Residence before leaving						
	Rural	98 (21.5)	358 (78.5)	1.0		
	Urban	157 (33.7)	309 (66.3)	1.86	[1.38, 2.49]	<0.01
Getting salary on time						
	Got all salary properly	78 (19.8)	316 (80.2)	1.0		
	Got salary some times	134 (37.1)	227 (62.9)	2.39	[1.72, 3.32]	<0.01
	Never got salary	13 (44.8)	16 (55.2)	3.29	[1.52, 7.13]	<0.01
Time for break						
	Yes	111 (26.4)	310 (73.6)	1.0		
	No	137 (28.0)	353 (72.0)	1.08	[0.81, 1.45]	0.59
Being allowed to visit friends/relatives						
	Allowed	84 (29.1)	205 (70.9)	1.0		
	Not allowed	144 (28.5)	362 (71.5)	0.97	[0.71, 1.34]	0.856
Being allowed to contact family						
	Allowed	153 (29.9)	359 (70.1)	1.0		
	Not allowed	72 (26.4)	201 (73.6)	0.84	[0.61, 1.17]	0.301
Health status at the time of return						
	Very bad	60 (37.0)	102 (63.0)	1.0		
	Somewhat bad	114 (44.0)	145 (56.0)	1.34	[0.89, 2.00]	0.158
	Same as before	50 (15.8)	266 (84.2)	0.32	[0.21, 0.50]	<0.01
	Very good	30 (16.8)	149 (83.2)	0.34	[0.21, 0.57]	<0.01
Knowledge of the culture of the destination country						
	Know much	16 (35.6)	29 (64.4)	1.0		
	Know some	90 (32.5)	187 (67.5)	0.87	[0.45, 1.69]	0.685
	Didn’t know at all	148 (24.6)	454 (75.4)	0.59	[0.31, 1.12]	0.186
Knowledge of the work supposed to do						
	Know much	26 (42.6)	35 (57.4)	1.0		
	Know some	119 (31.2)	262 (68.8)	0.61	[0.35, 1.06]	0.081
	Didn’t know at all	111 (22.7)	379 (77.3)	0.39	[0.23, 0.68]	<0.01
Knowledge of the life style in the destination country						
	Know much	9 (50.0)	9 (50.0)	1.0		
	Know some	98 (39.8)	148 (60.2)	0.66	[0.25, 1.73]	0.399
	Didn’t know at all	150 (22.5)	518 (77.5)	0.29	[0.11, 0.74]	<0.05

**Table 4:** Multivariable logistic regression analysis for the association of socio-demographic and migration related factors with CMD

Variables		Odds ratio	95% CI	*P* value
Religion				
	Orthodox	1.0		
	Muslim	0.60	[0.36, 0.98]	<0.05
	Protestant	3.46	[1.47, 8.17]	<0.01
	Catholic	4.90	[0.61, 39.46]	0.135
Region				
	Tigray	1.0		
	Amhara	2.03	[1.03, 4.02]	<0.05
	Oromia	2.01	[1.06, 3.78]	<0.05
	SNNPR	0.39	[0.18, 0.84]	<0.05
	Addis Ababa	1.74	[0.60, 5.07]	0.31
Marital status				
	Married	1.0		
	Never married	0.96	[0.65, 1.42]	0.838
	Divorced	1.16	[0.47, 2.84]	0.744
Residence before leaving				
	Rural	1.0		
	Urban	1.42	[0.93, 2.17]	0.102
Getting salary on time				
	Got all salary properly	1.0		
	Got salary some times	2.15	[1.42, 3.24]	<0.05
	Never got salary	2.55	[1.02, 6.71]	<0.05
Health status at the time of return				
	Very bad	1.0		
	Somewhat bad	1.40	[0.85, 2.31]	0.19
	Same as before	0.38	[0.22, 0.65]	<0.05
	Very good	0.26	[0.13, 0.52]	<0.05
Knowledge of the work supposed to do				
	Know much	1.0		
	Know some	0.60	[0.27, 1.34]	0.21
	Didn’t know at all	0.62	[0.27, 1.45]	0.27
Knowledge of the life style in the destination country				
	Know much	1.0		
	Know some	0.90	[0.20, 4.03]	0.89
	Didn’t know at all	0.53	[0.16, 2.44]	0.41

In the multivariable model (Table 4), Muslim religion (OR = 0.55; 95% CI = 0.40, 0.77), being from SNNPR (OR = 0.46; 95% CI = 0.29, 0.74) and current health status same as (OR = 0.32; 95% CI = 0.21, 0.50) or better than (OR = 0.34; 95% CI = 0.21, 0.57) it was before migration were negatively associated with CMD. Whereas being a follower of Protestant religion (OR = 3.46; 95% CI = 1.47, 8.17), being from Amhara (OR = 2.03; 95% CI = 1.03, 4.02) and Oromia (OR = 2.01; 95% CI = 1.06, 3.78) regions, and got salary sometimes (OR = 2.15; 95% CI = 1.42, 3.24) or not at all (OR = 2.55; 95% CI = 1.02, 6.71) in the destination were positively associated with CMD.

## Discussion

The prevalence of common mental disorders (CMD) in this population of migrant returnees from different Middle East countries and South Africa was found to be 27.6% (using a cut-off point of ≥8). We could not find previous large scale epidemiological studies on this population from Ethiopia to compare our findings with. Nevertheless, there are a few community-based studies that estimated the magnitude of CMD in the general population using the same measure as ours. These studies are summarized in Table 5 below for comparison.

**Table 5:** Studies in Ethiopia that estimated the prevalence of common mental disorder

Reference	Setting	Sample size	CMD measure	Cut-off	Prevalence
[41]	Urban	40	SRQ-20	≥5	12.0%
[42]	Urban (mothers only)	611	SRQ-20	≥11	9.8%
[43]	Rural	2000	SRQ-20	≥11	11.2%
[44]	Urban	10,203	SRQ-20	≥6	11.7%
[45]	Rural	10,468	SRQ-20	≥11	17.4%
[46]	Mixed (mothers only)	1400	SRQ-20	≥8	22.0%
[47]	Rural (antenatal mothers only)	1065	SRQ-20	≥5	12.0%
[48]	Rural (post-natal mothers only)	954	SRQ-20	≥5	4.6%

The prevalence of CMD we found in this study is higher than what has been reported in the general population in Ethiopia (both from rural and urban areas). The prevalence rates reported in these studies range from 5 to 22%, although different cut-off values were used. This suggests that migrant returnees are more likely to have symptoms related to CMD than the general population. Previous qualitative studies carried out in this population found that many Ethiopian migrant returnees from different Middle Eastern countries experience sexual, physical and emotional abuse, starvation, imprisonment, and difficulty adapting to a different culture [22]. Following these experiences, migrants reported such symptoms as headache, stomachache, irritability, suicidal thoughts, pessimism and sadness [23].

Studies conducted on domestic workers who migrated from Africa and South East Asia to the different Middle East countries found higher levels of psychiatric morbidity [17, 27]. For instance, El-Hilu and colleagues [16] found out that women from low-income countries who migrated to the Middle East as domestic workers experience psychiatric morbidity at rates two to five times higher than the native population. Abebaw’s [3] qualitative study also indicated that at the end of the complex oppression they went through, domestic workers experienced confusion and frustration which culminated in mental illness. Five of his 17 respondents narrated about the memory loss, fear, lack of sleep, frequent quarrel with family, day dreaming, shouting, hallucination, and other related mental health problems, including difficulty recognizing their family.

In this study, the CMD symptoms such as headache, poor appetite, problem sleeping, sadness and feeling nervous were reported by more than a third of the participants. On the other hand, symptoms like hand tremors, trouble thinking clearly, suicidal thought, difficulty making decisions and functional impairment were reported by less than 20% of the participants. These findings are consistent with previous qualitative studies conducted in Ethiopia among returnees from the different Middle East countries who migrated for domestic work [3, 22, 23]. The general tendency in Africa, and in Ethiopia in particular, is to express mental health problems in terms of somatic symptoms [34] (a headache, stomachache, problems sleeping and poor appetite). It must be noted, however, that the prevalence of suicidal ideation in this study is very high (15%) when we compare it with previous reports from Ethiopia. For instance, a recent large-scale study in rural Ethiopia conducted with both facility-based and population-based sample reported 12 month prevalence of suicidality of 6.3% among the community sample and 10.3% in the facility-based sample [35].

In the univariate models, being Muslim, being from SNNPR, and being a person of good current physical health were negatively associated with CMD; whereas being from the Addis Ababa or Oromia regions, divorced, of urban residence, having salary denied and a lack of awareness about the lifestyle and culture of the destination country were found to increase risk for CMD. The findings of this study showed that migration related stressors (such as being unable to get salary on time and salary denial) and preparation of the migrant beforehand (e.g. awareness about the culture, the lifestyle, food and religion of the destination country) are important predictors of CMD symptoms. Our finding is consistent with a number of previous studies conducted in North America [14, 36–38], Europe [11, 12, 28, 29, 39, 40] and the Middle East [16, 27] among migrants and refugees from different countries.

Being Muslim religion and being from SNNPR were identified as protective factors. The relationship of religion to migrant distress is a compelling finding that merits further discussion. Ethiopian Muslims have some connection and affiliation with people from Middle East countries due to religious, cultural and language similarity and therefore, they likely experience less stress during their stay in the destination country compared to Christians. Muslim migrants are also better prepared before their migration and have better awareness about the culture and lifestyle of Middle East countries than Christian migrants do. The majority of migrant returnees from SNNPR who participated in this study are Muslims and thus, presumably had a better chance to get prepared for the migration in terms of knowing the culture and way of life of the destination countries.

In the multivariable model, Muslim religion, being from SNNPR and good physical health status were found to be negatively associated with CMD symptoms independently; whereas Protestant religion, being from Amhara and Oromia regions and salary denial were found to be risk factors after adjusting for several confounders. Being divorced, living in urban residence, and knowledge of the work they are supposed to do and the lifestyle and culture of the destination country were not associated with CMD in the multivariable model. Since migrant returnees tend to express their psychological distress through somatic complaints, it is not surprising for good current physical health status to be a protective factor.

Significantly, Ethiopian migrants who are Christians and from Amhara, Addis Ababa and Oromia regions are likely to be strangers to the culture, religious practices, way of life, language and food in the Middle East. Therefore, they experience more abuse, conflict, imprisonment, starvation and other acculturation problems. Unable to get salary on time and salary denial were found to be important risk factors for developing CMD symptoms. The major push factor for Ethiopian migrants to head to the Middle East and South Africa is seeking a better paying job to be able to support their family. And when their expectations and dreams are frustrated by the difficult realities, they get stressed, nervous and hopeless. As a consequence, they will develop anxiety and depressive symptoms and even suicidal ideation and attempt. Abebaw [3] in his interview of Ethiopian trafficking returnee women documented that the young women expressed extreme anger and frustration related to salary denial or not getting salary on time more than physical and even sexual abuse. This is partly because they went with determination to endure the latter challenges (if they faced them) for improving their economic situation. So, salary denial is the cruelest injustice of all because many migrants have endured all of the other pain for their salary.

Overall, our study indicated that improved pre-migration characteristics, including preparation before migration (e.g. better understanding of the work at the destination, employment conditions, and legal issues), cross-cultural awareness, knowledge and skills may be determinants of mental distress than stressors encountered once residing in the destination country. Therefore, the concerned body in Ethiopia needs to design training and awareness creation programs for potential migrant workers. Central to this objective, defined standards and criteria (in terms of educational level, job related skills and knowledge and skills related to the language, culture, and legal and religious practices of the host community/country) need to be identified and put in place for those who want to migrate.

The following limitations should be taken into account when interpreting the findings of this study. The first and likely the most important limitation is that participants were not selected using probability sampling (as it was difficult to define a clear sampling frame owing to poor migrant data set in Ethiopia); we rather used availability and snowball sampling and this may result in sampling bias. Although several pre-migration, migration and post migration factors are associated with the symptoms of CMD, we were only able to measure selected pre-migration and post-migration characteristics. We particularly did not assess experiences of migrants while traveling to the destination country. We did not include migrant returnees who were admitted into hospital due to psychiatric or physical health conditions and therefore, the prevalence of CMD reported in this study is likely to be underestimated. Moreover, we did not validate the instruments we used to assess socio-demographic characteristics and migration related risk factors, albeit they were pilot tested and evaluated by experts.

## Conclusions

This study indicated that CMD symptoms are prevalent among Ethiopian migrant returnees from the Middle East and South Africa. Migration related factors such as less preparation for domestic work abroad, lack of awareness about the type of job they are supposed to do, low or no cross- cultural awareness and limited or no skill are important predictors of mental disorders in this population. Unable to fulfill aspirations and salary denial are also key risk factors for CMD symptoms. Overall the study indicated that pre-migration risk factors are associated with CMD. Therefore, better vocational, skill, and awareness creation training programs may help prevent some of these risk factors. A high proportion of migrant returnees have several mental health issues. Therefore, mental health care and rehabilitation services need to be expanded for this group of people.

Future research in this population should focus on estimating the prevalence of several mental disorders using probability sampling and diagnostic assessment instruments. Additional research should recognize the importance of follow-up with migrant returnees over time using longitudinal designs. A responsible body also needs to be designated for the proper management of data on migrant returnees. Today, Ethiopia has no agency or research institute charged with this responsibility. Sadly, therefore, much of the data available is a mere “guestimate” provided by various organizations in and outside of Ethiopia.

## Additional file

Additional file 1: English version of the questionnaire developed to assess socio-demographic and migration related characteristics. (DOCX 29 kb)

## Abbreviations

CMD: Common mental disorders
OR: Odds ratio
SNNPR: Southern Nations, Nationalities and People’s Region
SRQ: Self-reporting questionnaire
