# Supplementary material for: Prevalence and associated factors of common mental disorders among Ethiopian migrant returnees from the Middle East and South Africa
Source: BMC Psychiatry. 2017 Apr 19;17:144. doi: 10.1186/s12888-017-1310-6 (PMC5395750; doi:10.1186/s12888-017-1310-6)
Supplement: Additional file 1: — English version of the questionnaire developed to assess socio-demographic and migration related characteristics. (DOCX 29 kb) [file 12888_2017_1310_MOESM1_ESM.docx]

**Addis Ababa University**

**Office of the Vice President for Research and Technology Transfer**

**Thematic research on “Managing the Socio-cultural, Health, Legal and Economic Dimensions of Migration: Emphasis on Ethiopian Migrants in the Middle East and South Africa”**

**Questionnaire to assess socio-demographic and migration related characteristics**

**(To be administered to Ethiopian migrant returnees from the Middle East and South Africa)**

***NOTE: Information about the study and verbal consent form are available in separate sheets***

| **Part I: Socio-demographic related questions** | | | | |
| --- | --- | --- | --- | --- |
| **S.N** | **Question** | **Response categories** | **Codes** | |
| 1 | Sex | Male | 1 | |
|  |  | Female | 2 | |
| 2 | What is your age? | __________________years |  | |
| 3 | What is your religion? | Orthodox Christian | 1 | |
|  |  | Muslim | 2 | |
|  |  | Protestant Catholic | 3 | |
|  |  | Other, specify______________ | 4 | |
| 4 | What is your Ethnic group? (Write in the space provided in the right hand side of this question) | _________________________ | | |
| 5 | From which one of the following Regions are you from? | Tigrai | | 1 |
|  |  | Amahara | | 2 |
|  |  | Oromia | | 3 |
|  |  | Southern Nations, Nationalities and Peoples Region | | 4 |
|  |  | Addis Ababa | | 5 |
|  |  | Dire Dawa | | 6 |
| 6 | What is your educational level? | Can’t read and write | | 1 |
|  |  | Able to read and write | | 2 |
|  |  | First cycle primary (grade 1-4) | | 3 |
|  |  | Second cycle primary (grade 5-8) | | 4 |
|  |  | First degree and above | | 5 |
| 7 | What is your marital status? | Single | | 1 |
|  |  | Married | | 2 |
|  |  | Divorced | | 3 |
|  |  | Widowed | | 4 |
|  |  | Separated | | 5 |
| 8 | If your marital status is “married”, did you get married voluntarily? | Yes | | 1 |
|  |  | No | | 2 |
| 9 | If your marital status is “married”, how much satisfied are you in your marriage? | Very much satisfied | | 1 |
|  |  | Satisfied | | 2 |
|  |  | Not satisfied | | 3 |
| 10 | Do you have children? | Yes | |  |
|  |  | No | |  |
| 11 | If your response to question 10 above is “yes”, how many children do you have? | __________________ children | | |
| 12 | What was your occupation before migration? | Helping parents | | 1 |
|  |  | Housewife | | 2 |
|  |  | House maid | | 3 |
|  |  | Daily laborer | | 4 |
|  |  | Government employed | | 5 |
|  |  | Other, specify _______________ | | 6 |
| 13 | Were you living in a rural or urban area before migration? | Rural | | 1 |
|  |  | Urban | | 2 |
| 14 | From whom/where did you get information about your country of migration? | Broker | | 1 |
|  |  | Family | | 2 |
|  |  | Friend | | 3 |
|  |  | Media | | 4 |
| **Part II: Family background related questions** | | | | |
| 15 | What is the living arrangement of your family? | Live together | | 1 |
|  |  | Divorced | | 2 |
|  |  | One of the parents not alive | | 3 |
|  |  | Both parents not alive | | 4 |
| 16 | What is the educational status of the head of your household? | Can’t read and write | | 1 |
|  |  | Able to read and write | | 2 |
|  |  | First cycle primary (grade 1-4) | | 3 |
|  |  | Second cycle primary (grade 5-8) | | 4 |
|  |  | General secondary school (grade 9-10) | | 5 |
|  |  | Preparatory school (grade 11-12) | | 6 |
|  |  | Certificate or diploma | | 7 |
|  |  | First degree and above | | 8 |
| 17 | What is the occupation of your parents? | Farming | |  |
|  |  | Trading | |  |
|  |  | Government employed | |  |
|  |  | Other, specify ______________ | |  |
| **Part Three: Question related to migration** | | | |  |
| 18 | What was the country where you migrated? | _____________________________________ | | |
| 19 | By what means did you go to abroad? | Through Ministry of Labour and Social Affairs (through contract) | | 1 |
|  |  | By bus or through sea crossing borders | | 2 |
| 20 | What was/were the main reason/s for migration? | Poverty | | 1 |
|  |  | Being influenced by others | | 2 |
|  |  | To be self-sufficient | | 3 |
|  |  | Pressure from others | | 4 |
| 21 | Where does the highest pressure for migration come from? | Friends | | 1 |
|  |  | Family or relative | | 2 |
|  |  | Self | | 3 |
|  |  | Other, please specify | | 4 |
| 22 | How long is it since you returned from your migration? | ________________ months | | |
| 23 | What is your reason for returning? | To visit family | | 1 |
|  |  | Deported | | 2 |
|  |  | Was not comfortable for me there | | 3 |
|  |  | Finishing contract | | 4 |
| 24 | How long did you stay abroad? | _____________________ years | | |
| 25 | Do you have a relative in the country where you migrated? | Yes | | 1 |
|  |  | No | | 2 |
| 26 | How many people were you serving in the household you were employed? | _____________________ people | | |
| 27 | Were you receiving your salary regularly and properly when you were abroad? | Yes | | 1 |
|  |  | No | | 2 |
| 28 | How were you sending money to your family when you were abroad? | With people | | 1 |
|  |  | Using bank | | 2 |
|  |  | Through bank account | | 3 |
|  |  | Didn’t send money at all | | 4 |
| 29 | If you sent money, how it was spent? | Used by family for subsistence | | 1 |
|  |  | Family bought asset for me | | 2 |
|  |  | Family bought asset for itself | | 3 |
|  |  | Deposited in the bank | | 4 |
| 30 | If “deposited in the bank” for question 29 above, how did you spend it after you returned back? | ____________________________________ | | |
| 31 | On average, how many hours per day were you working abroad? | __________________ hours | | |
| 32 | Did you have weekly or monthly leave? | Yes | | 1 |
|  |  | No | | 2 |
| 33  34 | Were you allowed to go out of the home you were employed to visit your friends?  Did your employer allow you to communicate with your family through the phone? | Yes | | 1 |
|  |  | No  Yes | | 2  1 |
|  |  | No | | 2 |
| 35 | If “yes” to question 34, with what interval? | ___________________________ | | |
| 36 | Was your life abroad better than it was before you leave? | Yes, it was better | | 1 |
|  |  | It was the same | | 2 |
|  |  | No, it was worse | | 3 |
| 37 | Of the following, which of the problem (s) did you face abroad? (multiple response is possible) | Labor exploitation | | 1 |
|  |  | Sexual abuse | | 2 |
|  |  | Physical abuse/beating | | 3 |
|  |  | Verbal abuse/slander | | 4 |
|  |  | Not giving salary | | 5 |
|  |  | Undermining | | 6 |
|  |  | Denial of medication | | 7 |
|  |  | Withholding/confiscating of documents | | 8 |
| 38 | During your stay abroad, how good was the support you received from the agency? | Very good | | 1 |
|  |  | Good | | 2 |
|  |  | Weak | | 3 |
|  |  | Very weak | | 4 |
|  |  | Had no communication at all | | 5 |
| 39 | During your stay abroad, how good was the support you received from the embassy? | Very good | | 1 |
|  |  | Good | | 2 |
|  |  | Weak | | 3 |
|  |  | Very weak | | 4 |
|  |  | Had no communication at all | | 5 |
| 40 | How was your overall health condition at the time when you returned back? | Very bad | | 1 |
|  |  | Somewhat bad | | 2 |
|  |  | Same as before I went | | 3 |
|  |  | Very good | | 4 |
| 41 | What was the level of welcome you received from your family at the time when you returned back? | Very good | | 1 |
|  |  | Good | | 2 |
|  |  | Bad | | 3 |
| 42 | What is your future plan? | Going back | | 1 |
|  |  | Creating own job | | 2 |
|  |  | Waiting for what the government will do | | 3 |
|  |  | Waiting for what my family will do | | 4 |
|  |  | Other, specify_________________ | | 5 |
| 43 | Who is responsible for the harm Ethiopians are experiencing because of unsafe migration? | The migrants themselves | | 1 |
|  |  | Family | | 2 |
|  |  | Brokers | | 3 |
|  |  | The government | | 4 |
|  |  | Agencies | | 5 |
|  |  | Other, specify________________ | | 6 |
| 44 | Compared to your overall expectation before you go broad, how much did you get now? | More than my expectation | | 1 |
|  |  | Up to my expectation | | 2 |
|  |  | Less than my expectation | | 3 |
|  |  | I get nothing | | 4 |
| 45 | How many Ethiopians are engaged in commercial sex work after going abroad for the purpose of domestic work or other labor work? | Many | | 1 |
|  |  | Few | | 2 |
|  |  | None | | 3 |
| 46 | How good is the support you are receiving from the concerned government bodies after you returned back? | Very good | | 1 |
|  |  | Good | | 2 |
|  |  | Weak | | 3 |
|  |  | Very weak | | 4 |
| 47 | How was your skill of the language of your country of destination? | Very high | | 1 |
|  |  | Medium | | 2 |
|  |  | Low | | 3 |
| 48 | How was your skill of communication in your country of destination? | Very high | | 1 |
|  |  | Medium | | 2 |
|  |  | Low | | 3 |
| 49 | How different were the body languages (facial, hand, eyes, vocal etc) of your country here and your country of destination there? | Very high | | 1 |
|  |  | Medium | | 2 |
|  |  | Low | | 3 |
| 50 | How much problem did you face because you didn’t know the culture and language of your country of destination? | Very high | | 1 |
|  |  | Medium | | 2 |
|  |  | Low | | 3 |
| 51 | What was your level of knowledge about the duties, responsibilities and rights from the employers before you leave? | Very much | | 1 |
|  |  | Medium | | 2 |
|  |  | Nothing | | 3 |
| 52 | What was your level of knowledge about the culture of your country of destination before you leave? | Very much | | 1 |
|  |  | Medium | | 2 |
|  |  | Nothing | | 3 |
| 53 | What was your level of knowledge about the work you are expected to do in your country of destination before you leave? | Very much | | 1 |
|  |  | Medium | | 2 |
|  |  | Nothing | | 3 |
| 54 | What was your level of knowledge about the life style in your country of destination before you leave? | Very much | | 1 |
|  |  | Medium | | 2 |
|  |  | Nothing | | 3 |
| 55 | If you received training before you leave, was the training adequate? (If you didn’t receive any training, no need to respond to this question) | Yes, it was adequate | | 1 |
|  |  | No, it was not adequate | | 2 |
| 56 | How much did you pay to complete the process of migrating abroad? | _________________________ | |  |
| 57 | If you migrated through contract, how much did you know in detail about it? | Know very well | | 1 |
|  |  | Know some how | | 2 |
|  |  | Know nothing | | 3 |
| 58 | If you migrated legally through contract, was the contract respected by all stakeholders (employer, agency and employee)? | Yes, it was respected | | 1 |
|  |  | No, it was not respected | | 2 |
| 59 | If the contract was not respected, who violated the contract? | Employer | | 1 |
|  |  | Employee | | 2 |
|  |  | Agency | | 3 |
| 60 | When you were facing problems in the country where you were working, how much were you receiving legal support? | I was receiving enough | | 1 |
|  |  | I was receiving some what | | 2 |
|  |  | I was receiving nothing | | 3 |
